# Intermittent Theta Burst Stimulation Ameliorates Cognitive Deficit and Attenuates Neuroinflammation *via* PI3K/Akt/mTOR Signaling Pathway in Alzheimer’s-Like Disease Model

**DOI:** 10.3389/fnagi.2022.889983

**Published:** 2022-05-17

**Authors:** Andjela Stekic, Milica Zeljkovic, Marina Zaric Kontic, Katarina Mihajlovic, Marija Adzic, Ivana Stevanovic, Milica Ninkovic, Ivana Grkovic, Tihomir V. Ilic, Nadezda Nedeljkovic, Milorad Dragic

**Affiliations:** ^1^Laboratory for Neurobiology, Department of General Physiology and Biophysics, Faculty of Biology, University of Belgrade, Belgrade, Serbia; ^2^Department of Molecular Biology and Endocrinology, Vinča Institute of Nuclear Sciences, National Institute of the Republic of Serbia, University of Belgrade, Belgrade, Serbia; ^3^Medical Faculty of Military Medical Academy, University of Defence, Belgrade, Serbia; ^4^Institute for Medical Research, Military Medical Academy, Belgrade, Serbia

**Keywords:** intermittent theta burst stimulation, Alzheimer’s disease, trimethyltin, neurodegeneration, cognitive deficit, neuroinflammation, Akt/Erk/mTOR signaling

## Abstract

Neurodegeneration implies progressive neuronal loss and neuroinflammation further contributing to pathology progression. It is a feature of many neurological disorders, most common being Alzheimer’s disease (AD). Repetitive transcranial magnetic stimulation (rTMS) is a non-invasive stimulation which modulates excitability of stimulated brain areas through magnetic pulses. Numerous studies indicated beneficial effect of rTMS in several neurological diseases, including AD, however, exact mechanism are yet to be elucidated. We aimed to evaluate the effect of intermittent theta burst stimulation (iTBS), an rTMS paradigm, on behavioral, neurochemical and molecular level in trimethyltin (TMT)-induced Alzheimer’s-like disease model. TMT acts as a neurotoxic agent targeting hippocampus causing cognitive impairment and neuroinflammation, replicating behavioral and molecular aspects of AD. Male Wistar rats were divided into four experimental groups–controls, rats subjected to a single dose of TMT (8 mg/kg), TMT rats subjected to iTBS two times per day for 15 days and TMT sham group. After 3 weeks, we examined exploratory behavior and memory, histopathological and changes on molecular level. TMT-treated rats exhibited severe and cognitive deficit. iTBS-treated animals showed improved cognition. iTBS reduced TMT-induced inflammation and increased anti-inflammatory molecules. We examined PI3K/Akt/mTOR signaling pathway which is involved in regulation of apoptosis, cell growth and learning and memory. We found significant downregulation of phosphorylated forms of Akt and mTOR in TMT-intoxicated animals, which were reverted following iTBS stimulation. Application of iTBS produces beneficial effects on cognition in of rats with TMT-induced hippocampal neurodegeneration and that effect could be mediated *via* PI3K/Akt/mTOR signaling pathway, which could candidate this protocol as a potential therapeutic approach in neurodegenerative diseases such as AD.

## Introduction

Neurodegeneration is a complex pathological event characterized by a progressive loss of nerve cells and deterioration of neural functions, underlying many neurological disorders, most common being Alzheimer’s disease (AD) (Erkkinen et al., 2018). Neurodegenerative disorders are often accompanied by neuroinflammatory activation of astrocytes and microglia, which transit from a physiological, quiescent state to a reactive phenotype, releasing various inflammatory factors thus actively contributing to further neuronal degeneration (Colonna and Butovsky, 2017; Brambilla, 2019). There is no known effective drug/treatment for any neurodegeneration and for many diseases the clear cause has not been fully discovered (Dugger and Dickson, 2017), potentiating the need for experimental and therapeutic approaches. One experimental approach that mimics behavioral and histopathological aspects of hippocampal degeneration seen in AD is trimethyltin-induced neurodegeneration (Geloso et al., 2011). Trimethyltin (TMT) is a potent neurotoxicant specifically targeting human and animal limbic systems, particularly hippocampal formation (Balaban et al., 1988). Animals exposed to a single dose of TMT develop a series of symptoms, including seizures, hyperactivity, hyperexcitability, aggression, and severe cognitive deficit as a consequence of neuronal death of CA3/CA1 pyramidal neurons (Balaban et al., 1988; Trabucco et al., 2009; Corvino et al., 2015; Dragić et al., 2019; Park et al., 2019). Neuronal damage begins 2–4 days post-exposure, primarily in medial and proximal CA3 region and CA1 and can be detected at the end of the first week. It progressively worsens over the next 3 weeks (Dragić et al., 2021b) when almost entire medial and proximal CA3 and CA1 have degenerated. Neuronal loss is accompanied by early activation of astrocytes and microglia leading to sustained astrocyte-derived neuroinflammation (Dragić et al., 2021a,b) and microgliosis (Koczyk and Oderfeld-Nowak, 2000; Dragić et al., 2021b). TMT activates pathogenic pathways resulting in excitotoxicity, oxidative stress, mitochondrial dysfunction, intracellular calcium overload, and gene expression associated with apoptosis and necrosis (Little et al., 2012; Corvino et al., 2013; Lattanzi et al., 2013). Experimental data confirmed the significant roles of these mechanisms in the pathogenesis of human neurodegenerations, especially of Alzheimer’s disease, making TMT-induced degeneration a useful and translatable model convenient for probing diverse neuroprotective strategies (Park et al., 2011, 2019; Corvino et al., 2013; Jung et al., 2013). Repetitive transcranial magnetic stimulation (rTMS) is a safe, non-invasive neuromodulatory technique based on stimulation of cortical structures *via* electromagnetic pulses which are administered in a predefined protocol-dependent pattern. Stimulation at a certain frequency may lead to prolonged and increased cortical excitability after the period of stimulation (Thomson et al., 2020), affecting both neuronal and glial physiology (Cullen and Young, 2016). rTMS has already found use in clinical treatment for neurological and psychiatric diseases showing a beneficial effect in patients with drug-resistant depression (De Risio et al., 2020), motor symptoms of Parkinson’s disease (Nardone et al., 2019), and stroke (León Ruiz et al., 2018). It has been reported that rTMS, especially in combination with cognitive training, could be effective for improving mild-to-moderate cognitive decline in AD (Rabey and Dobronevsky, 2016). However, the full cellular and molecular mechanisms underlying these effects are largely unknown and the need for more comprehensive clinical trials still exists. Previous studies have shown that rTMS can attenuate reactive gliosis (Dragic et al., 2020), reduce neuronal apoptosis by regulating the expression of Bcl-2 and Bax protein family members (Uzair et al., 2022), restore pathological downregulation/induce activation of certain signaling pathways including mTOR (Yang et al., 2021), PI3K/Akt (Hou et al., 2021), modulate the biochemical environment against oxidative-nitrogen stress at a distance from the area of stimulation (Stevanovic et al., 2020) and affect cellular and molecular mechanisms underlying different forms of synaptic plasticity such as long-term potentiation and long-term depression (Fujiki et al., 2020). Observed neuroprotective effects have put rTMS on a list of promising therapeutic approaches in the treatment of neurodegenerative disorders such as AD, but very few studies on experimental models of neurodegenerations have been performed up to date. The aim of this study was to examine effects of intermittent theta burst stimulation (iTBS), an rTMS paradigm, on different aspects of TMT-induced hippocampal neurodegeneration regarding changes in behavior and cognition, pro- and anti-inflammatory markers, oxidative stress parameters, potentially important signaling pathways which could regulate abovementioned processes. iTBS is a form of rTMS protocol which has been shown to produce similar if not greater effects on brain activity than standard rTMS. Its major advantage is reduction in administration duration and consistency in application across literature (Chung et al., 2015). Results in this field of study are crucial for understanding the mechanisms underlying iTBS effects, finding a new potential cellular and molecular treatment targets of this paradigm of rTMS which could candidate it as a new therapeutic approach in treatment of neurodegenerative disorders.

## Materials and Methods

### Animals

A total of 54 2-month-old male *Wistar* rats, bred in the animal facility at Centre of Veterinary Service, Ministry of Defence, Serbia, were used in this study. Animals were housed (3–4 per cage) under the following conditions: 12 h light/dark regime, constant ambient temperature 23 ± 2°C and humidity, food and water *ad libitum*. All experimental procedures were approved by the Ethical Committee of Vinča Institute of Nuclear Sciences (Application No. 323-07-02057/2017-05) in compliance with EU Directive 2010/63/EU.

### Treatment and Experimental Groups

Animals were randomly assigned into four experimental groups: Control (*n* = 12), TMT (*n* = 15), TMT + iTBS (*n* = 12), TMT + iTBSsh (*n* = 15). On day 0, animals of the TMT, TMT + iTBS and TMT+ iTBSsh group received a single intraperitoneal injection of TMT (8 mg/kg, administered in the volume of 1 mL 0.9% saline). Control group received an adequate volume of 0.9% saline solution. The animals were monitored daily for 3 weeks, and sacrificed by decapitation (Harvard apparatus, Holliston, MA, United States).

### Theta Burst Stimulation Protocol

**FIGURE 1:**
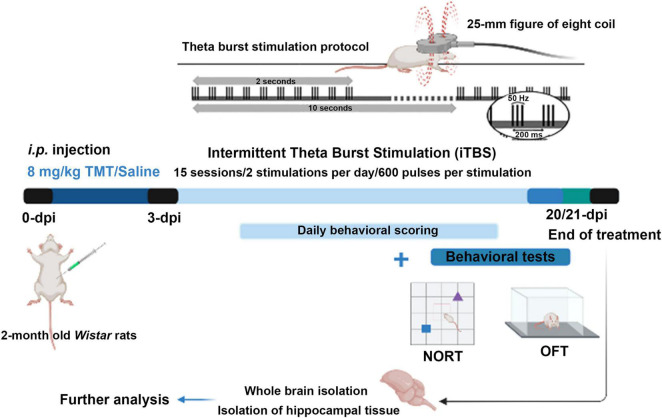
Experimental design diagram. On day 0, animals of the TMT, TMT + iTBS, and TMT + iTBS sham group received a single intraperitoneal injection of TMT (8 mg/kg dissolved in 0.9% saline). The control group received an adequate volume of saline solution. The animals were daily scored for signs of unusual behavior for 3 weeks. Intermittent theta-burst stimulation (iTBS) was applied on 3-dpi using MagStim Rapid^2^ device and a 25-mm figure-of-eight coil. Applied iTBS protocol consisted of 20 trains of ten bursts (3 pulses at a frequency of 50 Hz), repeated at 5 Hz (10 s intervals between trains, total duration of the procedure is 192 s), twice a day for 15 days in total. On the 20-/21-dpi, the behavior was evaluated by using the open field test (OFT) and novel object recognition (NORT) tests. After behavioral tests, animals were sacrificed. Whole brains and hippocampal tissue were isolated for immunohistological and immunoblot analyses.

Three days after intoxication intermittent protocol of theta-burst stimulation (iTBS) was applied. We chose this particular time point as it coincides with the onset of TMT-induced symptoms (Kaur and Nehru, 2013; Dragić et al., 2019). The stimulation was performed by MagStim Rapid^2^ device and a 25-mm figure-of-eight coil (MagStim Company, Whitland, United Kingdom). Applied iTBS protocol consisted of 20 trains of ten bursts (3 pulses at a frequency of 50 Hz), repeated at 5 Hz (10 s intervals between trains, with the total duration of the procedure of 192 s). Stimulation intensity (stimulator output) was set at 33%, which was just below/around the motor threshold value (Dragic et al., 2020). The motor threshold value was defined as a stimulus intensity which induces a minimal visible motor response of treated animals, most usually manifested as repetitive movement of mandible muscles mimicking chewing. The iTBS protocol did not induce any visible behavioral responses or distress to animals. Animals were gently held during the stimulation process, while left to move freely during 10-s intervals between trains. The stimulation was applied by holding the center of the coil gently above the frontal cranial bone, in close contact with the scalp. TMT + iTBS sham group (TMT + iTBSsh) was subjected to noise artifact – cage with two animals was set next to the MagStim Rapid^2^ device and the rats were allowed to listen to the sound of the stimulation followed by handling manipulation similar to TMT + iTBS group (Figure 1).

### Assessment of Behavioral and Aggression Severity Score

Aggressive behavior was monitored in freely moving rats and aggression severity score was determined as reported and described previously (Kaur and Nehru, 2013; Dragić et al., 2019). Briefly, every day (at 8:00 am) animals in a clean cage with bedding were scored according to the 1–4 scale, during a 2-min interval. The scores were as follows: (1) without symptoms, (2) shies from hand when grasped, mild tremor (3) avoids hand by running, struggles when captured or both, systemic tremor (4) leaps, struggles and bites when captured. Several animals in the TMT and TMT + iTBSsh group developed epileptic seizures during scoring, mostly triggered by sound (Supplementary Video 1) and repetitive rotational movements when captured by the tail (Supplementary Video 2), while in TMT + iTBS group those events were observed only once during 3 weeks.

### Open Filed and Object Recognition Test

**FIGURE 2:**
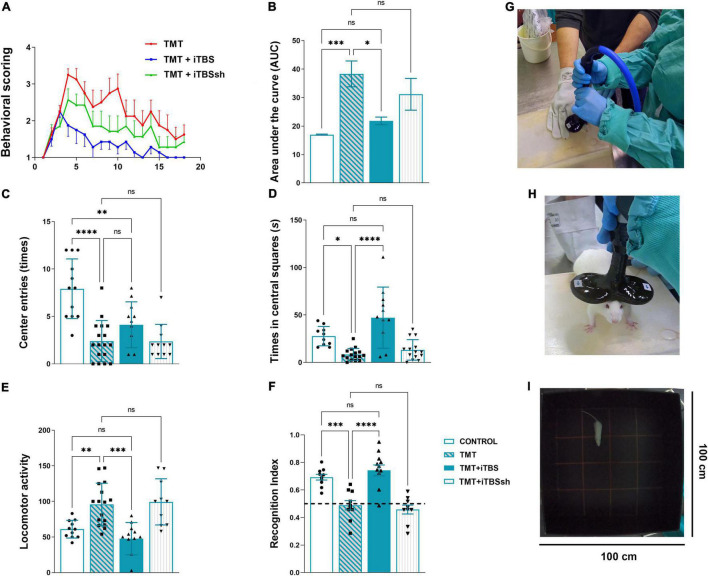
Effect of iTBS treatment on behavior of TMT rats. **(A)** Graphic and **(B)** quantitative analysis of behavioral animal scoring. Bars represent the mean AUC ± SD. **(C,D)** Anxiety-like behavior and **(E)** motor activity, assessed in the open-field test. Bars represent **(C)** a mean number of entries, **(D)** a mean duration spent in central squares (s), and **(E)** a mean total movements, ±SD. **(F)** Recognition index determined by the novel object recognition test. Bars represent mean recognition index ± SD. Interrupted line represents 50% of recognition index, where animals showed no discrimination between novel and familiar object. Results of *post hoc* Tukey’s test and significance shown inside graphs, *p < 0.05, ^**^p < 0.01, ^***^p < 0.001, ^****^p < 0.0001, ns, no significance. **(G,H)** iTBS coil placement. **(I)** The behavioral testing arena. Dots in the graphs represent values of individual animals.

The observation of spontaneous behavior was evaluated by the open field test (OFT). Animals were transferred to the behavior analysis room on the last day of the experiment and left for habituation to the environment for 2 h before the analysis. The behavioral observation room was completely separated from the cages to prevent acoustic and/or optic disturbance of the animals. All materials that have been in contact with the tested animals were cleaned with 70% ethanol thereafter to prevent any olfactory cues. The animals received one 5-min session by placement in the center of the empty black arena (100 × 100 × 50 cm, Figure 2I) to habituate them to the apparatus. The total movement was continuously recorded. Rat activity during this period was analyzed for spontaneous, exploratory activity in the open field test (OFT). Following the OFT and habituation with the apparatus, animals were placed in the center of the arena, at an equal distance from two identical rectangular objects (uniformly yellow-colored, diameter 5 cm, height 20 cm), left to freely explore for 5 min period, and returned to their home cages (sampling phase). After each animal, both arena and objects were thoroughly cleaned with 70% alcohol to ensure complete removal of any olfactory cures which could interfere with the test. After the 1 h-delay, animals were put in the center of the arena, at equal distance from objects, with one of the familiar objects being replaced by a new conical object (uniformly red-colored, diameter 15 cm, height 20 cm) and left to explore freely for another 5 min (testing phase). The time spent with each object was set as a baseline criterion, provided that sniffing, climbing and exploration of the object lasted more than 2 s which was considered as an active exploration (animals that did not meet these criteria were omitted from the analysis). Analysis of the sampling phase was also performed to examine whether animals showed preference toward any of two identical objects (Supplementary Figure 1). Recognition index (RI) represents the percentage of time spent at the novel object in respect to the total time spent at both objects (Antunes and Biala, 2012). Two researchers, blind of treatment groups, analyzed the behavioral results.

### Brain Tissue Preparation for Histological Techniques

Brains were carefully removed from the skull (*n* = 3–4/treatment group), fixed in 4% paraformaldehyde (PFA), cryoprotected and dehydrated in sucrose (10, 20, and 30%) in 0.2 M phosphate buffer pH 7.4 as described previously (Dragić et al., 2019). 25-μm thick coronal slices were cut on the cryostat, air-dried at room temperature (RT), and kept at −20°C until use.

### Histochemical and Immunohistochemical Staining

The coronal sections were stained with thionine and micrographs were taken on Leitz light microscope equipped with a Leica DFC320 camera. For each experimental group, several sections at different stereotaxic anterior-posterior (AP) coordinates were taken. Immunofluorescent staining procedures were performed as described previously (Dragić et al., 2021b). Briefly, sections were washed in PBS, blocked in 5% normal donkey serum, incubated with primary antibodies (Table 1), overnight at 4°C. The next day, sections were washed in PBS, incubated with appropriate secondary antibody for 2 h at RT, and mounted with Mowiol medium. Micrographs were taken by a confocal laser scanning microscope (LSM 510, Carl Zeiss, GmbH, Jena, Germany), with Ar multi-line (457, 478, 488, and 514 nm), HeNe (543 nm), HeNe (643 nm) laser at 40× and 63× (×2 digital zoom) DIC oil objectives, 40× and monochrome AxioCam ICm 1 camera (Carl Zeiss, GmbH, Jena, Germany).

**TABLE 1:** List of used primary and secondary antibodies.

Antibody	Source and type	Used dilution	Manufacturer
GFAP	Rabbit, polyclonal	1:500^IF^	DAKO, Agilent Z0334, RRID:AB_10013382
C3	Goat, polyclonal	1:300^IF^, 1:1000^WB^	Thermo Fisher Scientific PA1-29715 RRID: AB_AB_2066730
IL-10	Goat, polyclonal	1:100^IF^	Santa Cruz Biotechnology, sc-1783, RRID: AB_2125115
IL-1β/IL-1F2	Goat, polyclonal	1:100^IF^	R&D Systems, AF-501-NA, RRID: AB_354508
Bax	Rabbit, polyclonal	1:1000^WB^	Cell Signaling, #2772, RRID: AB_10695870
Bcl-2	Rabbit, polyclonal	1:1000^WB^	Cell Signaling #2876, RRID: AB_2064177
PI3K (p85)	Rabbit, monoclonal	1:500^WB^	Abcam, ab40755, RRID: AB_777258
p-Akt	Rabbit, polyclonal	1:1000^WB^	Cell Signaling, #9271S, RRID: AB_329825
t-Akt	Rabbit, polyclonal	1:1000^WB^	Cell Signaling, #9272S, RRID: AB_329827
p-mTOR	Rabbit, polyclonal	1:1000^WB^	Cell Signaling, #5536, RRID: AB_10691552
t-mTOR	Rabbit, polyclonal	1:1000^WB^	Cell Signaling, # 2983, RRID: AB_2105622
p-ERK 1/2	Rabbit, polyclonal	1:1000^WB^	Cell Signaling, #9101, RRID: AB_331646
t-ERK 1/2	Rabbit, polyclonal	1:1000^WB^	Cell Signaling, #9102, RRID: AB_330744
β-actin	Mouse, HRP-conjugated	1:5000^WB^	Abcam, ab49900, RRID: AB_867494
Anti-goat IgG Alexa Fluor 488	Donkey, polyclonal	1:400^IF^	Invitrogen A-11055, RRID: AB_142672
Anti-rabbit IgG Alexa Fluor 555	Donkey, polyclonal	1:400^IF^	Invitrogen A-21428, RRID:AB_141784
Goat anti-rabbit IgG, HRP-conjugated	Goat, polyclonal	1:30000^WB^	Abcam, ab6721, RRID: AB_955447

*WB, western blot; IF, immunofluorescence.*

### Image Analysis and Semi-Quantification

Hippocampal regions of interest (CA1, mCA3, and hilus/DG region) were captured at the same region levels (ranging from AP −2.70 to −5.30 mm) and under the same conditions (40× magnification, 1024 × 1024, laser gain and exposure). The images (10–16/animal) were analyzed with ImageJ software (free download from https://imagej.net/Downloads). Correlation and interdependency between red-green channels were estimated by calculating Pearson’s correlation coefficient (PCC). PCC values range from +1 (two fluorescent channels which are perfectly, linearly related) to −1 (two fluorescent channels which are perfectly, but inversely related). Values near 0 reflect distribution of two probes that are uncorrelated with one another (Dunn et al., 2011). The number of cells expressing the protein of interest was counted in the high-power field (HPF), based on an unequivocally visible cellular body with a few thicker branches and co-localization with analyzed protein. Immunoreactivity of the green signal was obtained by measuring fluorescence intensity in arbitrary units (AU). All manual quantification was performed by two independent researchers, blind of treatments. All three parameters were calculated from micrographs obtained at 40× magnification for each hippocampal region of every experimental group.

### Tissue Isolation and Western Blot Analysis

Hippocampal membrane and cytosolic fractions were separated using Subcellular Protein Fractionation Kit for Tissues (Cat. No. 87790, Thermo Fisher Scientific, Waltham, MA, United States), according to the manufacturer’s instructions. Briefly, homogenization of the frozen tissue samples was performed by a handheld homogenizer (Ultra-Turrax, Sigma-Aldrich, St. Louis, MO, United States) in an ice-cold cytoplasmic extraction buffer containing protease and phosphatase inhibitors. Supplied tissue strainers were used to filter tissue homogenate and obtained filtrates were centrifugated at 500 × *g* (Sorvall SL-50 T Super T21, Thermo Fisher Scientific, Waltham, MA, United States) for 5 min at 4°C. Cytoplasmic fraction remaining in supernatant was collected and the pellet was resuspended in an ice-cold membrane extraction buffer containing protease and phosphatase inhibitors, incubated on ice for 10 min and centrifuged at 3,000 × *g* for 5 min at 4°C to obtain membrane fraction remaining in supernatant after centrifugation. Protein concentration was determined using Pierce*™* BCA Protein Assay Kit (Cat. No. 23225, Thermo Fisher Scientific, Waltham, MA, United States) according to the manufacturer’s instructions. Western blot analyses (20–30 μg of sample proteins, *n* = 4–5 animals/group) were performed as previously described (Mitrović et al., 2017; Adzic and Nedeljkovic, 2018). Briefly, all samples were diluted in 6 × Laemmli buffer [4% sodium dodecyl sulfate (SDS), 0.02% bromophenol blue, 20% glycerol, 125 mmol/L Tris–HCl] and electrophoresis was run on a 10% SDS-polyacrylamide gel and proteins were transferred to PVDF membrane (0.45 mm, Millipore, Germany) using Trans-Blot^®^ Turbo*™* Transfer System (Cat. No. 1704150, Bio-Rad, Hercules, CA, United States) according to the manufacturer’s instructions. Membranes were blocked in 5% non-fat dry milk (Cat. No. 42590.01, SERVA, Germany) in Tris-buffered saline containing 0.1% Tween-20 (TBST), incubated overnight at 4° C with appropriate primary antibody in TBST (Table 1), rinsed in TBST and incubated with adequate horseradish-peroxidase (HRP)-conjugated secondary antibody in TBST (Table 1) using SmartBolt apparatus. Chemiluminescent signals were detected using ECL solution (Bio-Rad, Hercules, CA, United States) by ChemiDoc-It Imager (Ultra-Violet Products Ltd., Cambridge, United Kingdom). Primary and secondary antibodies were removed by stripping protocol using mild-stripping buffer (pH 2.2) containing 0.2 mmol/L glycine, 0.1% SDS and 1% Tween-20^1^ one time, so additional target protein could be blotted on the same membrane. Optical density (OD) of the specific band and actin band in each lane were quantified using the ImageJ program^2^, and the ratio in each lane was expressed relative to the control, arbitrarily defined as 100% ± SEM, from *n* = 2–4 technical replicates.

### Measurement of the Oxidative Stress

Oxidative stress was assessed by measuring several parameters in appropriate hippocampal fraction (*n* = 4–5 animals/group) as described in detail elsewhere (Stevanovic et al., 2020). Briefly, total superoxide dismutase activity (tSOD) was assessed by spectrophotometric determination of spontaneous epinephrine autooxidation decrease rate at 480 nm and expressed as units per milligram of total protein (U/mg). One unit is defined as an amount of enzyme required for 50% inhibition of epinephrine autooxidation (Sun and Zigman, 1978). Levels of free O_2_^•–^ were determined by reaction based on the O_2_^•–^ mediated reduction of nitroblue tetrazolium to monoformazan (μmol/mg protein), which is measured spectrophotometrically at 550 nm (Kono et al., 1997). Malondialdehyde (MDA) was quantified spectrophotometrically as a measurement of colored pigment formed after incubation with TBA reagent (water solution of 15% trichloroacetic acid and 0.375% TBA) at 95°C at pH 3.5. Absorbance was measured at 532 nm and results were expressed as μmol/mg protein (Girotti et al., 1991). Levels of NO were evaluated from the deproteinized samples and determined by directly measuring nitrite concentrations spectrophotometrically at 492 nm, and nitrates were converted into nitrites by cadmium reduction (Navarro-Gonzálvez et al., 1998). Total sulfhydryl groups were spectrophotometrically measured at 412 nm by Ellman’s method (Ellman, 1959). Total glutathione levels (GSH + ½ GSSG, in GSH equivalents) were determined by a DNTB-GSSG reductase recycling assay. The rate of 5-thio-2-nitrobenzoic acid (TNBA) formation, which is proportional to the total glutathione concentration, was measured spectrophotometrically at 412 nm and the results were expressed as μmol/mg of proteins.

### Statistical Analysis

All data were analyzed for normality and appropriate parametric statistical tests were used. One-way analysis of variance (One-way ANOVA) was performed for statistical comparison between groups, followed by Tukey’s *post hoc* test for multiple comparisons between experimental groups. All values are presented as mean ± SD or SEM as indicated in Figure legends. The values of *p* < 0.05 were considered statistically significant. For all analysis and graphical presentation GraphPad Prism 9.0 (San Diego, CA, United States) software package was used. Results of *post hoc* tests are described in detail in figure legends.

## Results

### Intermittent Theta Burst Stimulation Significantly Reduced Trimethyltin-Induced Hyperactivity, Aggressive Behavior, and Tremor

Animals were scored daily for signs of the “TMT syndrome,” which include hyperactivity, aggressive behavior, and tremor (Figure 2A). TMT-treated animals exhibited first symptoms on the 3-day post-intoxication (dpi), which progressed over 3 weeks with several peaks and partial recoveries (Figure 2A, red line). We have chosen 3-dpi to start iTBS stimulation as it was the time point of the onset of the first symptoms. Control animals did not exhibit any unusual behavior and kept a score of 1 for 3 weeks (*X*-axis value). Animals treated with iTBS (Figure 2A, blue line) showed progressive and uninterrupted improvement over the 3 weeks, did not show any aggressive behavior from 6-dpi, and expressed only mild tremor, which subsided in the final days of the experiment. TMT + iTBSsh group (Figure 2A, *green line*) presented symptoms similar to TMT group. The total score for each experimental group was obtained by averaging individual scores determined as the area under the curve (AUC) and the means were analyzed by One-way ANOVA (*F*_3,51_ = 7.95, *p* < 0.0001, Figure 2B). Significant difference was obtained for the comparison between TMT and TMT + iTBS (*p* < 0.05) TMT (*p* < 0.001), and iTBSsh and control (*p* < 0.01), whereas no differences were found detected between intact control group and TMT + iTBS.

### Intermittent Theta Burst Stimulation Reduces Trimethyltin-Induced Hyperactivity and Anxiety-Related Behavior

The open-field test is used to evaluate the effects of iTBS on TMT-induced hyperactivity and anxiety-related behavior (Figure 2). Significant changes were observed regarding anxiety-related behavior, which is expressed as the number of entries (Figure 2C, *F*_3,46_ = 14.66, *p* < 0.0001) and the time spent in the central quadrants of the arena (Figure 2D, *F*_3,46_ = 12.37, *p* < 0.0001). iTBS-treated animals showed increased number in both entries and time spent in central quadrants (Figures 2C,D). Changes in general locomotor activity was also observed following TMT intoxication and iTBS treatment (Figure 2E, *F*_3,46_ = 10.48, *p* < 0.0001). Following TMT intoxication, rats exhibited increased locomotion, while iTBS treatment reverted it to control levels (Figure 2E).

### Intermittent Theta Burst Stimulation Improves Trimethyltin-Induced Cognitive Impairment

The effect of iTBS treatment on TMT-induced cognitive impairment was assessed by the object recognition test (Figure 2F). No difference was observed in exploration time and the number of approaches to the objects during the sampling phase (Supplementary Figure 1). A significant difference was observed during the test phase (*F*_3,42_ = 19.35, *p* < 0.0001), which suggested a notable improvement of impaired cognitive abilities in iTBS-treated animals after TMT intoxication (Figure 2F).

### Intermittent Theta Burst Stimulation Reduced Trimethyltin-Induced Neuronal Death and Inflammation

**FIGURE 3:**
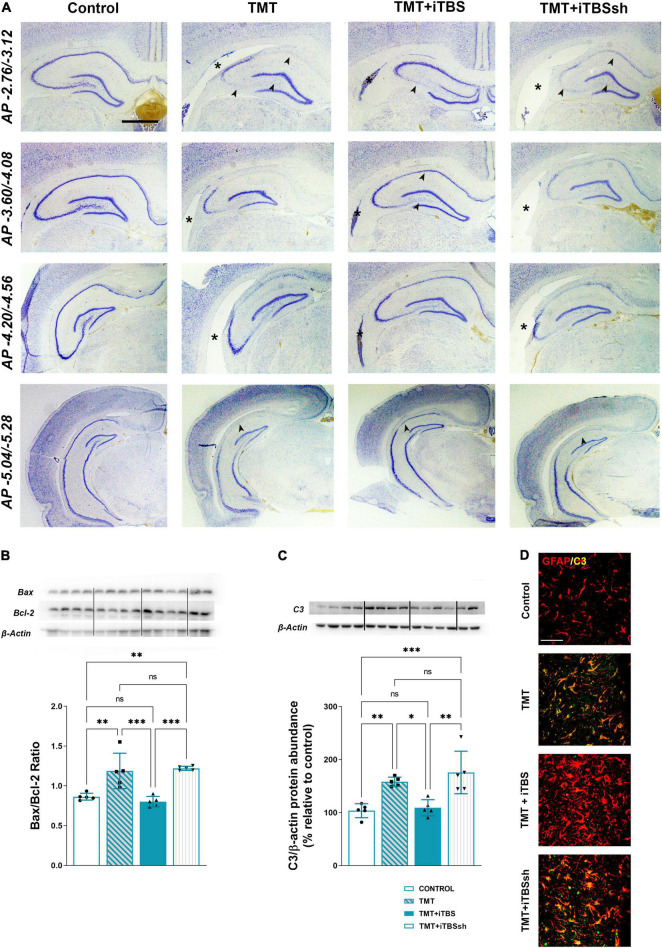
Effect of iTBS treatment on TMT-induced neuronal death and inflammation. **(A)** Thionine-stained coronal sections at different stereotaxic anterior-posterior (*AP*) coordinates. Arrowhead indicates thinned neuronal cell layers, while asterisk marks the position of the lateral ventricle for a comparison. Scale bar = 500 μm, applies to all micrographs. Representative membrane and semi-quantitative Western blot analysis showing **(B)** relative Bax/Bcl-2 and **(C)** C3 protein abundance, expressed relative to control, arbitrary defined as 100%. Bars in **(B)** represent the ratio between Bax and Bcl-2 relative abundance, determined as mean OD ratio of the specific protein band to actin band for each group ± SEM (from *n* = 5 individual animals, in 2–4 technical replicates). Bars in **(C)** represent the mean OD of the C3 band and actin band in each group normalized to control (arbitrarily defined as 100%) ± SEM (from *n* = 5 individual animals, in 2–4 technical replicates). Results of *post hoc* Tukey’s test and significance shown inside graphs: *p < 0.05, ^**^p < 0.01, ^***^p < 0.001, ^****^p < 0.0001, ns, no significance. Dots in the graphs represent values of individual animals. **(D)** Double immunofluorescent staining of coronal hippocampal sections captured in the same region levels directed to GFAP (*red*) and C3 (*green*). Scale bar = 50 μm.

The overall changes in the hippocampal cytoarchitecture were assessed with the use of Nissl histological staining (Figure 3A). Animals that received iTBS treatment (Figure 3A, third column) showed significantly less neuronal death in respect to TMT and iTBSsh, which exhibited conspicuous neurodegeneration in CA1, medial CA3 (mCA3), and the hilar region of the dentate gyrus (hilus/DG), and extended lateral ventricles. The changes observed at the histological level were confirmed by determining the Bax/Bcl-2 abundance in the cytosolic fractions (*F*_3,16_ = 16.62 *p* < 0.0001), which pointed toward increased Bax/Bcl-2 levels in TMT and TMT + iTBS sham groups, and near the control level in TMT + iTBS group (Figure 3B). iTBS treatment also reverted to near control level protein expression of complement 3 (C3), which was markedly increased in TMT and iTBSsh (Figure 3C, *F*_3,16_ = 12.29 *p* < 0.0001). C3 was confined to reactive astrocytes, as previously shown (Figure 3D; Dragić et al., 2021b). Since no differences were observed between TMT and TMT + iTBSsh group at behavioral, histopathological and molecular, it has been excluded from further figures.

### Intermittent Theta Burst Stimulation Alters Trimethyltin-Induced Astrocytic Inflammation

**FIGURE 4:**
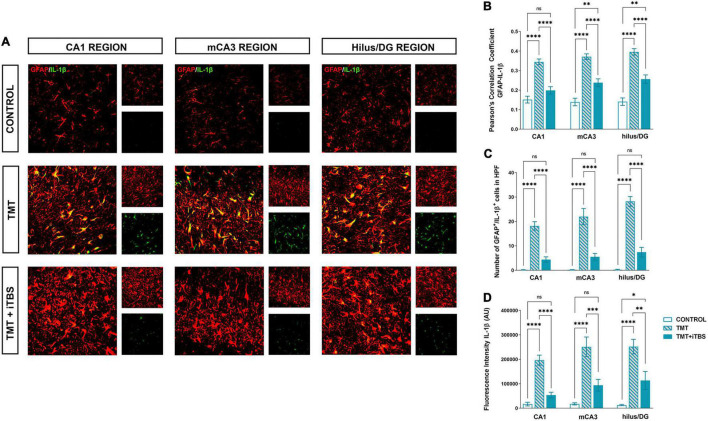
Effect of iTBS treatment on TMT-induced astrocytic inflammation. **(A)** Double immunofluorescence staining directed to GFAP (*red*) and IL-1β (*green*) of coronal hippocampal sections captured in the same region levels. Scale bar = 50 μm. Integration of immunofluorescence intensity and quantitative analyses showing **(B)** Pearson’s correlation coefficient (PCC), **(C)** number of GFAP^+^/IL-1β^+^ cells in high power field (HPF), and **(D)** fluorescence intensity of IL-1β in arbitrary units (AU). Bars represent the mean value ± SEM of specific parameters calculated from acquired images (10–16/animal). Results of *post hoc* Tukey’s test and significance shown inside graphs: *p < 0.05, ^**^p < 0.01, ^***^p < 0.001, ^****^p < 0.0001, ns, no significance.

**FIGURE 5:**
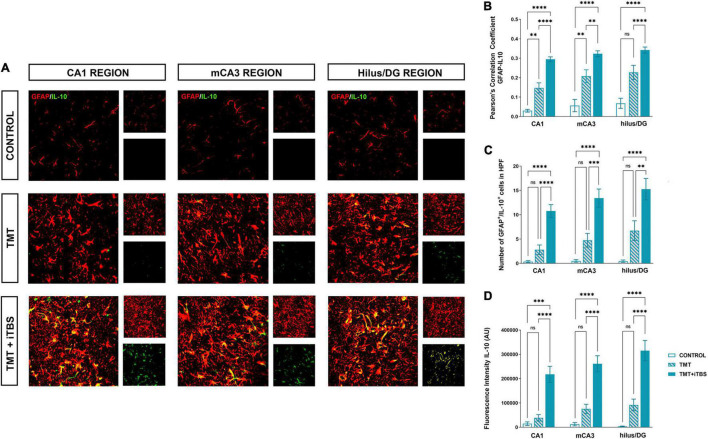
Effect of iTBS treatment on the inflammatory status of reactive astrocytes. **(A)** Immunofluorescence staining directed to GFAP (*red*) and IL-10 (*green*) of coronal hippocampal sections captured in the same region levels and. Scale bar = 50 μm. Integration of immunofluorescence intensity and quantitative analyses showing **(B)** Pearson’s correlation coefficient (PCC), **(C)** number of GFAP^+^/IL-10^+^ cells in high power field (HPF), and **(D)** fluorescence intensity of IL-10 in arbitrary units (AU). Bars represent the mean values ± SEM of specific parameters calculated from all acquired images (10–16/animal). Results of *post hoc* Tukey’s test and significance shown inside graphs: *p < 0.05, ^**^p < 0.01, ^***^p < 0.001, ^****^p < 0.0001, ns, no significance.

Trimethyltin induces pronounced astrocytic activation and release of IL-1β or IL-10 (Dragić et al., 2021b). Treatment with iTBS did not alter the morphology of GFAP^+^ cells but reduced the TMT-induced increase in IL-1β-immunoreactivity (*ir*) (Figure 4A). To evaluate regional changes of IL-1β expression we calculated PCC (Figure 4B), the number of GFAP^+^/IL-1β^+^ astrocytes (Figure 4C), and the total intensity of the IL-1β fluorescence signal (Figure 4D). We found that TMT significantly increased all examined parameters in all three regions when compared to control, while iTBS treatment reduced the inflammation to levels similar to control, although astrocytes remained reactive phenotype (Table 2). The increase in all examined parameters measuring co-localization of GFAP and IL-10 was observed following iTBS treatment, especially in the hilus/DG (Figure 5 and Table 3).

**TABLE 2:** Results of one-way ANOVA analysis.

	Region	ANOVA results	Tukey’s *post hoc* test		
			Control vs. TMT	TMT vs. TMT + iTBS	Control vs. TMT + iTBS
PCC analysis	CA1	*F*_(2,47)_ = 36.23, *p* < 0.0001	*p* < 0.0001	*p* < 0.0001	ns
	mCA3	*F*_(2,48)_ = 40.76, *p* < 0.0001	*p* < 0.0001	*p* < 0.0001	*p* < 0.01
	Hilus/DG	*F*_(2,47)_ = 43.18, *p* < 0.0001	*p* < 0.0001	*p* < 0.0001	*p* < 0.01
Number of GFAP^+^/IL-1β^+^ cells in HPF	CA1	*F*_(2,46)_ = 36.32, *p* < 0.0001	*p* < 0.0001	*p* < 0.0001	ns
	mCA3	*F*_(2,48)_ = 36.04, *p* < 0.0001	*p* < 0.0001	*p* < 0.0001	ns
	Hilus/DG	*F*_(2,45)_ = 97.70, *p* < 0.0001	*p* < 0.0001	*p* < 0.0001	ns
Fluorescence intensity IL-1β	CA1	*F*_(2,46)_ = 41.43, *p* < 0.0001	*p* < 0.0001	*p* < 0.0001	ns
	mCA3	*F*_(2,47)_ = 16.22, *p* < 0.0001	*p* < 0.0001	*p* < 0.001	ns
	Hilus/DG	*F*_(2,46)_ = 17.10, *p* < 0.0001	*p* < 0.0001	*p* < 0.01	*p* < 0.05

**TABLE 3:** Results of one-way ANOVA analysis.

	Region	ANOVA results	Tukey’s *post hoc* test		
			Control vs. TMT	TMT vs. TMT + iTBS	Control vs. TMT + iTBS
PCC analysis	CA1	*F*_(2,46)_ = 32.24, *p* < 0.0001	*p* < 0.0001	*p* < 0.0001	*p* < 0.01
	mCA3	*F*_(2,49)_ = 17.41, *p* < 0.0001	*p* < 0.01	*p* < 0.01	*p* < 0.0001
	Hilus/DG	*F*_(2,46)_ = 13.99, *p* < 0.0001	ns	*p* < 0.0001	*p* < 0.0001
Number of GFAP^+^/IL-10^+^ cells in HPF	CA1	*F*_(2,47)_ = 18.69, *p* < 0.0001	ns	*p* < 0.0001	*p* < 0.0001
	mCA3	*F*_(2,48)_ = 29.69, *p* < 0.0001	ns	*p* < 0.0001	*p* < 0.0001
	Hilus/DG	*F*_(2,47)_ = 17.85, *p* < 0.0001	ns	*p* < 0.01	*p* < 0.0001
Fluorescence intensity IL-10	CA1	*F*_(2,46)_ = 28.13, *p* < 0.0001	ns	*p* < 0.0001	*p* < 0.0001
	mCA3	*F*_(2,47)_ = 20.51, *p* < 0.0001	ns	*p* < 0.001	*p* < 0.0001
	Hilus/DG	*F*_(2,45)_ = 15.86, *p* < 0.0001	ns	*p* < 0.01	*p* < 0.0001

### Intermittent Theta Burst Stimulation Reduces Trimethyltin-Induced Oxidative Stress

**FIGURE 6:**
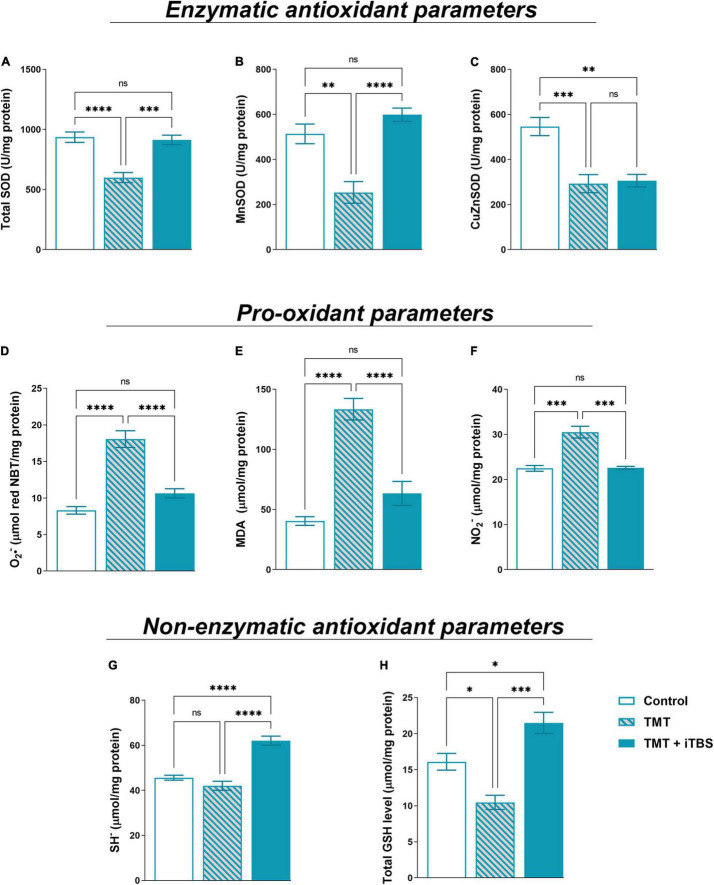
Effect of iTBS treatment on TMT-induced oxidative stress. Spectrophotometric analysis of antioxidant parameters: **(A)** total SOD, **(B)** MnSOD, **(C)** CuZnSOD, and pro-oxidant parameters: **(D)** O_2_^•–^, **(E)** MDA (malondialdehyde), **(F)** NO_2_^–^ and non-enzymatic antioxidant parameters- **(G)** SH^–^ and **(H)** total GSH level, measured in hippocampal protein homogenates (*n* = 4–5 animals/group). Bars represent mean activity, expressed as U/mg protein **(A–C)**, μmol/mg protein **(D–H)**, ±SEM. Results of *post hoc* Tukey’s test and significance are shown inside graphs: *p < 0.05, ^**^p < 0.01, ^***^p < 0.001, ^****^p < 0.0001, ns, no significance.

Trimethyltin intoxication is accompanied by changes in oxidative status which is the driving factor of neurodegeneration and neuroinflammation (Kang et al., 2016). iTBS reverted TMT-induced reduction in tSOD activity (Figure 6A, *F*_2,17_ = 18.92, *p* < 0.0001) and reverted MnSOD activity (Figure 6B, *F*_2,16_ = 21.00, *p* < 0.0001) to near control levels. The treatment had no impact on TMT-induced decrease in CuZnSOD activity (Figure 6C, *F*_2,16_ = 14.42, *p* < 0.001). The levels of pro-oxidative parameters O^2–^, MDA and NO_2_^–^ were examined (Figures 6D–F). The enhanced levels of free O^2–^ (Figure 6D, *F*_2,16_ = 39.52, *p* < 0.0001), MDA (Figure 6E, *F*_2,16_ = 39.52, *p* < 0.0001) and NO_2_^–^ (Figure 6F, *F*_2,18_ = 36.62, *p* < 0.0001) following TMT intoxication were reverted by iTBS to near control level. Finally, we examined two-non enzymatic antioxidant parameters – levels of SH^–^ and total GSH content (Figures 6G,H). In TMT-treated animals, levels of SH^–^ did not change, but iTBS significantly increased SH level (Figure 6G, *F*_2,17_ = 37.86, *p* < 0.0001). iTBS also overcompensated the TMT-induced reduction of total GSH for about 50% in respect to control (Figure 6H, *F*_2,17_ = 21.39, *p* < 0.001).

### Intermittent Theta Burst Stimulation Rescues Trimethyltin-Attenuated PI3K/Akt/mTOR and ERK1/2 Signaling

**FIGURE 7:**
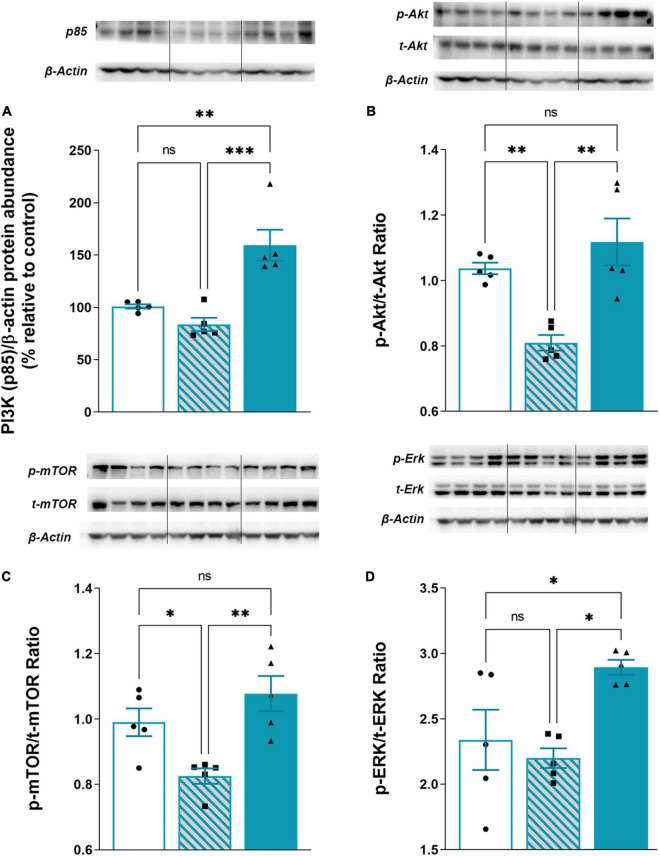
Effect of iTBS treatment on TMT-attenuated PI3K/Akt/mTOR signaling. Representative support membrane and quantitative data of Western blot analysis showing relative protein abundance of **(A)** PI3K (p85) or phosphorylated/non-phosphorylated **(B)** p-Akt/t-Akt ratio, **(C)** p-mTOR/t-mTOR, **(D)** p-ERK/t-ERK ratio in the hippocampal cytosol. Bars represent mean value of target protein **(A)** or ratio of phosphorylated and non-phosphorylated forms **(B–D)** normalized to β-actin abundance ± SEM (from *n* = 4–5 individual animals, in 2–4 technical replicates), expressed relative to control (arbitrary defined as 100%). Results of *post hoc* Tukey’s test and significance are shown inside graphs: *p < 0.05, ^**^p < 0.01, ^***^p < 0.001, ^****^p < 0.0001, ns, no significance. Dots in the graphs represent values of individual animals.

PI3K/Akt/mTOR and ERK1/2 signaling have a critical role in neurodegenerative/neuroinflammatory (Lu and Malemud, 2019). Therefore we investigated changes in protein expression of PI3K/Akt/mTOR and ERK1/2 in cytosolic fraction following TMT-induced neurodegeneration and iTBS treatment (Figure 7). The expression of the regulatory subunit of PI3K (p85) was slightly decreased in TMT animals, whereas iTBS significantly increased the expression (Figure 7A, *F*_2,12_ = 17.94, *p* < 0.001). The downstream signaling protein kinase B/Akt showed a marked decrease in p-Akt/t-Akt following TMT, while iTBS treatment reverted it to the levels similar to control (Figure 7B, *F*_2,12_ = 12.81, *p* < 0.01). A significant reduction in phosphorylation levels of mTOR was found after TMT-induced neurodegeneration, which was restored following iTBS treatment (Figure 7C, *F*_2,12_ = 9.35, *p* < 0.01). iTBS significantly increased levels of phosphorylated ERK 1/2 form, while TMT remained without the effect (Figure 7D, *F*_2,12_ = 6.52, *p* < 0.05).

## Discussion

In the present study, we evaluated the potential therapeutic use of iTBS protocol on a behavioral, histopathological, and molecular level in the Alzheimer’s-like disease model induced by TMT intoxication. Acute TMT intoxication results in a well-described model of the neurodegeneration in the hippocampus and associated limbic and cortical regions (Balaban et al., 1988; Dragić et al., 2019, 2021b), and consequent cognitive impairment. That makes a model a perfect tool for studying diverse neuroprotective strategies (Koda et al., 2008; Urano and Tohda, 2010; Park et al., 2011; Kang et al., 2016), particularly in AD as the model replicates some key behavioral and molecular features of this disease (Nilsberth et al., 2002; Andjus et al., 2009; Geloso et al., 2011; Sadoughi, 2019). Given that behavioral changes occur in the initial stages after TMT exposure, they might be used to follow the efficacy of novel therapeutic strategies (Moser, 2011). We used a battery of neurobehavioral tests to screen behavior in non-stressful (activity monitoring in a cage) and a stressful environment (open field and novel object recognition test). Behavioral changes in a non-stressful environment manifested as tremor, hyperactivity and aggression became apparent at 3-dpi and peaked at 4-dpi, which is in agreement with previous data (Kaur and Nehru, 2013; Dragić et al., 2019). Thereafter, behavioral scores in TMT and iTBSsh animals spontaneously declined toward partial recovery, with two lower peaks at 10*-*dpi and 14-dpi, indicating ongoing neuronal degeneration (Geloso et al., 2011). The next set of neurobehavioral tests assessed animal behavior in stressful environments using OF and NORT after 21-dpi, when hippocampal neurodegeneration was shown to be at peak (Geloso et al., 2011; Dragić et al., 2021b). Animals exhibited anxiety-like behavior and hyperlocomotion, which are typical for TMT-induced hippocampal neurodegeneration (Geloso et al., 2011; Kang et al., 2016). Since TMT-induced hippocampal degeneration results in severe cognitive deficits (Park et al., 2011, 2019; Jung et al., 2013; Kang et al., 2016), we tested short-term memory by using NORT. As anticipated, animals exposed to TMT showed a severe reduction in recognition index demonstrating impairment in processes involved in memory retention (Botton et al., 2010; Antunes and Biala, 2012). The neurotoxin primarily affects CA3 pyramidal neurons and partially CA1 pyramidal neurons, thus severing the anatomical connections between the entorhinal cortex – dentate gyrus and hippocampus proper (Balaban et al., 1988; Geloso et al., 2004, 2011). Disruption of these excitatory/inhibitory connections critical for information flow in hippocampal formation is most likely responsible for aggression, hyperactivity, hyperexcitability, and cognitive impairment (Geloso et al., 2011; Lee et al., 2016). The behavioral data were corroborated with histopathology which showed extensive loss of CA3 and CA1 pyramidal neurons and consequent ventricular dilatation (Balaban et al., 1988; Koczyk and Oderfeld-Nowak, 2000; Andjus et al., 2009). As previously shown, main pro-apoptotic and anti-apoptotic markers, as well as markers of inflammation (C3, IL-1β), were dramatically increased, the latter being restricted to astrocytes as the main source of inflammatory factors (Corvino et al., 2015; Marchese et al., 2018; Dragić et al., 2021a,b). Another consequence of massive neuronal death such as that seen after TMT intoxication inevitably leads to increased production of reactive oxidative and nitrosative species (Kaur and Nehru, 2013). We demonstrated significant disturbance in pro-oxidative and anti-oxidative parameters following TMT intoxication, which are in agreement with previous results (Kaur and Nehru, 2013; Dragić et al., 2021a). Oxidative stress has been implicated in TMT-induced apoptosis and inhibition of PI3K/Akt/mTOR signaling pathways, which are important regulators of growth and cell survival as well as in the memory process (Selvaraj et al., 2012; Zhao et al., 2016). Accordingly, we found a significant reduction in phosphorylated forms of Akt and mTOR, which are also seen in other models of toxin-induced neurodegeneration (Selvaraj et al., 2012; Zhou et al., 2016) and are associated with cognitive impairment (Palumbo et al., 2021).

To the best of our knowledge, this is the first study to demonstrate that iTBS treatment notably improves motor behavior, emotional expression, cognitive abilities, and histopathological and inflammatory status after TMT-induced neurodegeneration, although some beneficial effects on cognition have been demonstrated for other rTMS protocols in animal models (Tan et al., 2013; Guo et al., 2017) and human subjects (Yin et al., 2020). One of the main problems with various rTMS paradigms is that it hugely varies pre-set parameters such as machine output strength, length of session, duration of application which makes it difficult to compare obtained results. iTBS paradigm is a powerful excitatory protocol of rTMS with consistent parameters in literature and with equal or higher efficacy with reduced time of exposure (Chung et al., 2015), which candidates it as an excellent approach in both human and animal studies. In our study, animals stimulated with iTBS had less severe symptoms with a much shorter period without seizures and aggression. Following repeated iTBS, the animals expressed less frequent and milder symptoms of “TMT syndrome,” probably due to the beneficial effects on hippocampal formation, which is involved in all aspects of the behavioral expression of emotions, including aggressiveness (Vertes, 2006). Our results showed that animals stimulated with iTBS had normal locomotor behavior and reduced anxiety levels, which was previously shown in animal models of epilepsy and post-traumatic stress disorder with the use of rTMS stimulation protocols (Wang et al., 2015, 2019). It has been suggested that rTMS induces neuronal plasticity, modulates neurotransmitter and neurotrophic factors leading to LTP-like changes in stimulated areas (Uzair et al., 2022), thus improving cognitive processes (Heath et al., 2021). Prolonged stimulation with iTBS could evoke similar mechanisms and alter neuronal excitability and levels of neurotrophic factors, thus improving cognitive status in the TMT model of neurodegeneration. The cognitive and behavioral improvements can be explained by iTBS-induced changes at a cellular level. Namely, iTBS reduced apoptotic cell death in mCA3 and CA1 sectors and attenuated astrocyte-driven inflammation. Previous data demonstrate that rTMS prevents neuronal death by inhibiting several members of the Bcl-2 family, particularly pro-apoptotic factors Bax, Bad, and Bcl-xS (Guo et al., 2017; Uzair et al., 2022), which are the key mediators in TMT-induced apoptosis as well [for review see Geloso et al. (2011)]. Attenuated neuronal death following iTBS was accompanied by a reduction in pro-inflammatory factors and an increase in anti-inflammatory factors. Anti-inflammatory effects of rTMS have been demonstrated in neurological disorders and animal models (Aftanas et al., 2018; Hong et al., 2020; Clarke et al., 2021; Dragić et al., 2021c). Furthermore, rTMS was found to inhibit the polarization of astrocytes toward neurotoxic (A1-like) phenotype (Hong et al., 2020), which are also present in TMT-induced neurodegeneration (Dragić et al., 2021a,b). Reduced expression of inflammatory factors could be a result of reduced neuronal death following iTBS, but also a result of the direct effect of iTBS on glial cells (Cullen and Young, 2016; Cullen et al., 2019). It has been shown that rTMS affects astrocytes both *in vitro* and *in vivo*, implicating astrocytes as cellular effectors of rTMS (Cullen and Young, 2016; Clarke et al., 2021). Inflammatory phenomena are tightly coupled with the overproduction of reactive oxygen and nitrogen species, which may produce neuronal degeneration. Improvement in oxidative status following iTBS, and its antioxidative potential (Medina-Fernández et al., 2018; Stevanovic et al., 2020) could be mediated *via* nuclear factor (erythroid-derived 2)-like 2 (Nrf2) that is involved in transcriptional regulation of antioxidative enzymes (Jaiswal, 2004). Previous studies reported that TMS increases the expression of Nrf2 in neuroinflammatory conditions (Tasset et al., 2013; Liang et al., 2021), which could also be the mechanism in TMT-mediated oxidative stress. To dissect molecular changes that could underlie favorable effects of iTBS, we investigated PI3K/Akt/mTOR and ERK 1/2 signaling as key players in the regulation of several processes, including proliferation, apoptosis, learning, and memory (Xu et al., 2020). An increase in all these kinases could be put in perspective of reduction of TMT-induced anxiety-like behavior and improvement of cognitive functions following iTBS. It has been shown that *Akt2* knockout and heterozygote mice exhibit anxiety-like behavior and impaired hippocampal-dependent learning (Palumbo et al., 2021), emphasizing the role of Akt and its downstream targets in these processes. Thus, an iTBS-induced increase in phosphorylated Akt could be a significant contributor to the reversal of selective behavioral impairments seen after TMT intoxication. Furthermore, mTOR, as a downstream target of Akt and ERK 1/2, is directly implicated in protein synthesis and involved in the process of learning and memory (Bekinschtein et al., 2007; Palumbo et al., 2021). An increase in phosphorylated mTOR and improved cognitive performance have been observed in the pharmaco-resistant model of depression following iTBS (Lee et al., 2021). Numerous studies have demonstrated that rTMS induces an increase in BDNF [for review see Uzair et al. (2022)] and the receptor tyrosine-related kinase B (TrkB) (Lee et al., 2021), both of which are reduced in TMT-induced neurodegeneration (for review see (Geloso et al., 2011)). Therefore, some of beneficial effects of iTBS may be mediated through BDNF-TrkB signaling. Increased release of BDNF and enhanced TrkB signaling may be responsible for the induction of PI3K/Akt/mTOR and favorable effects on inflammation, apoptosis, anxiety-related behavior, and cognitive improvement seen in the present study after iTBS (Stevanovic et al., 2019; Lee et al., 2021; Uzair et al., 2022). At the end, although the results of present study demonstrate significant beneficial effects of iTBS, it is noteworthy to mention some technical limitations which concern the size and manual placement of the coil, which do not allow focal stimulation of specific cortical areas; therefore the effects induced by iTBS stimulation may be the result of stimulation of both cortical and subcortical structures and their interconnections. Furthermore, due to this technical limitation it is not possible to conclude definitive mechanism and/or structures fully or partially involved in the recovery observed in this experimental paradigm. However, this approach may provide information about the potential effects of stimulation of deeper subcortical structures, which are inaccessible in human subjects.

## Conclusion

To the best of our knowledge, this is the first study demonstrating the beneficial effects of iTBS protocol on behavioral and cognitive performance and hippocampal cytoarchitecture in the Alzheimer’s-like disease model. Specifically, we found significantly reduces neuronal death, inflammation, and oxidative stress, reduced hyperactivity, aggressive behavior, and tremor, and improved cognitive status in TMT animals stimulated with iTBS protocol. Among critical signaling pathways, we demonstrated that iTBS rescued PI3K/Akt/mTOR signaling, which acts in favor of cell survival and recovery. Therefore, iTBS protocol as a paradigm of rTMS may be an excellent candidate for efficient, painless and non-invasive therapy of neurodegenerative disorders associated with cognitive deficits such as Alzheimer’s disease.

## Data Availability Statement

The original contributions presented in the study are included in the article/Supplementary Material, further inquiries can be directed to the corresponding author/s.

## Ethics Statement

The animal study was reviewed and approved by the Ethical Committee of Vinča Institute of Nuclear Sciences.

## Author Contributions

AS: methodology, validation, formal analysis, investigation, and writing – original draft. MZ: methodology, visualization, formal analysis, and writing – review and editing. MK and IG: methodology, formal analysis, resources, and writing – review and editing. KM, MA, IS, and MN: methodology, formal analysis, and writing – review and editing. TI: supervision, resources, and writing – review and editing. NN: project administration, supervision, funding acquisition, resources, and writing – review and editing. MD: conceptualization, methodology, validation, visualization, formal analysis, investigation, and writing – original draft. All authors contributed to the article and approved the submitted version.

## Conflict of Interest

The authors declare that the research was conducted in the absence of any commercial or financial relationships that could be construed as a potential conflict of interest.

## Publisher’s Note

All claims expressed in this article are solely those of the authors and do not necessarily represent those of their affiliated organizations, or those of the publisher, the editors and the reviewers. Any product that may be evaluated in this article, or claim that may be made by its manufacturer, is not guaranteed or endorsed by the publisher.

## Footnotes

1
https://www.abcam.com/protocols/western-blot-membrane-stripping-for-restaining-protocol

2
https://imagej.nih.gov/ij/
