# Supplementary material for: Intermittent Theta Burst Stimulation Ameliorates Cognitive Deficit and Attenuates Neuroinflammation via PI3K/Akt/mTOR Signaling Pathway in Alzheimer’s-Like Disease Model
Source: Front Aging Neurosci. 2022 May 17;14:889983. doi: 10.3389/fnagi.2022.889983 (PMC9152158; doi:10.3389/fnagi.2022.889983)
Supplement: Supplementary file 1 [file Image_1.pdf]

Supplementary Material

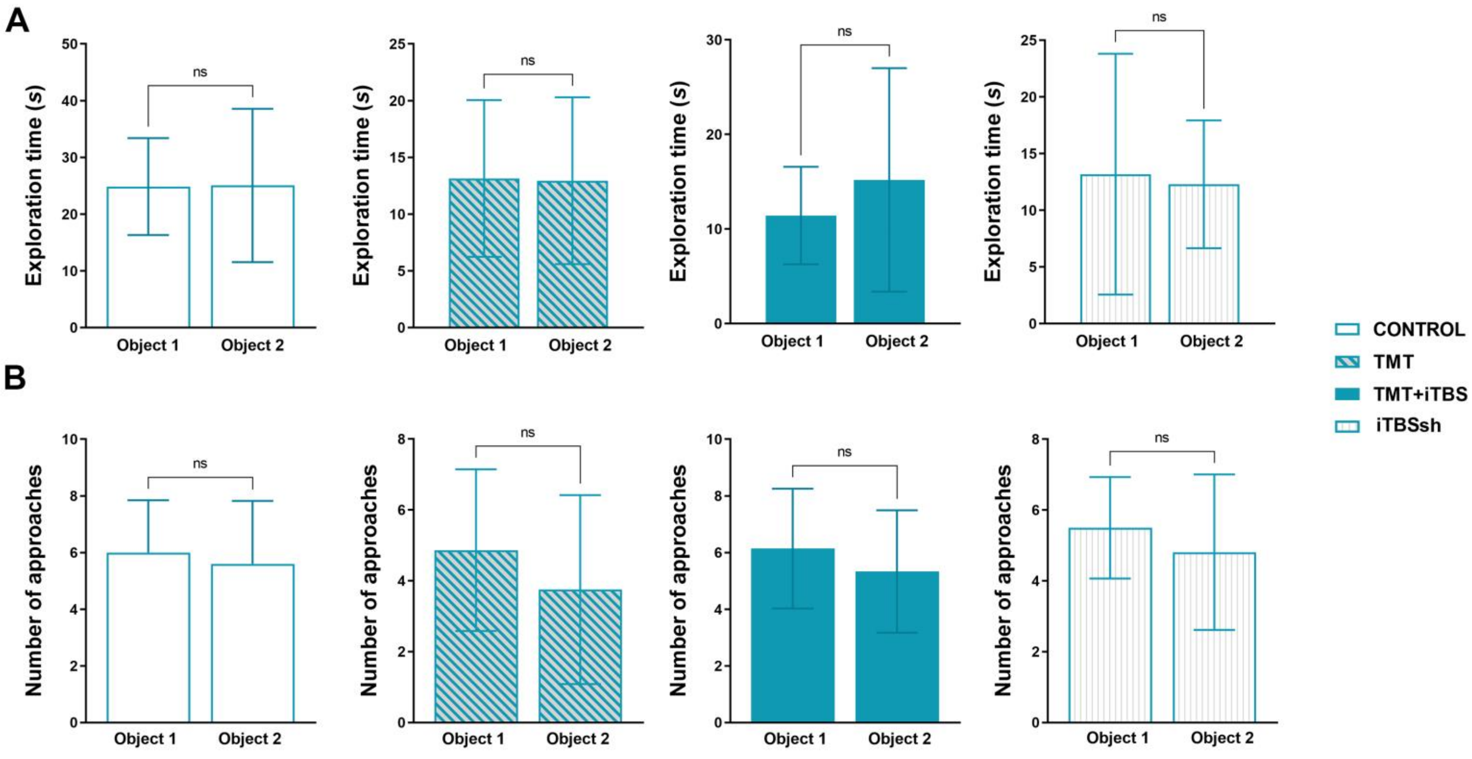

Supplementary Figure 1. Results of sample phase of Novel object recognition test

(**A)** Exploration time (*s*) rats spent at two identical objects named Object 1 and Object 2. (**B**) Number of approaches to Object 1 and Object 2. No statistical difference was observed in all examined group in both exploration time and number of approaches. Results of *post hoc* Tukey's test and significance shown inside graphs, **p < 0.05, ** p < 0.01, *** p < 0.001, **** p < 0.0001, ns- no significance.*
